# Co‐Producing the Design Brief for an Intervention to Increase Breast Cancer Awareness Among Black Women in Ante‐Natal Settings: A Qualitative Study With Breast Cancer Survivors, Community Members, and Midwives in the UK

**DOI:** 10.1111/hex.70936

**Published:** 2026-10-16

**Authors:** Afrodita Marcu, Lorraine Marke, Emma Ream, Jo Armes

**Affiliations:** ^1^ School of Health Sciences University of Surrey Guildford Surrey UK; ^2^ Patient and Public Involvement representative London UK

**Keywords:** behaviour change, Black women, breast cancer awareness, breast cancer inequalities, co‐production

## Abstract

**Background:**

Women of Black African/Caribbean heritage in the UK are more likely to develop breast cancer at an early age (before being eligible for breast screening from 50 years of age), be diagnosed with advanced breast cancer, and have lower survival than White British women. Culturally acceptable interventions are needed to encourage timely symptomatic presentation.

**Objective:**

Using the Behaviour Change Wheel (BCW) stages of intervention development, we developed the design brief for an intervention to be delivered in ante‐natal settings to Black women not yet eligible for breast cancer screening.

**Design/Methods:**

Four co‐production sessions with 14 women: 4 with a history of breast cancer, 5, without, and 5 healthcare professionals (4 midwives and 1 public health apprentice). One Patient and Public Involvement (PPI) representative of Black African & White heritage helped facilitate the sessions. The data were analysed deductively using Template Analysis and the themes were mapped onto the BCW stages and steps of intervention development.

**Results and Discussion:**

The participants agreed that the key behaviour to address is timely help‐seeking for breast changes indicative of breast cancer. It was agreed the intervention should have three main functions: education, that is, increase Black women's knowledge of breast cancer symptoms (*psychological capability*), training, that is, teach breast self‐checks (*physical capability*), and persuasion, that is, convince that a breast cancer diagnosis is not a death sentence and increase intentions to seek timely help (*reflective motivation*).

**Conclusion:**

Delivering breast cancer education in ante‐natal settings was seen as acceptable and feasible by the co‐production participants. Further feasibility and acceptability testing is needed.

**Patient or Public Contribution:**

A female breast cancer survivor of mixed Black African and White heritage was involved as PPI representative during the project. She advised on the draft funding application, the ethics documents, and the planning of each co‐production session. She helped recruit participants, facilitated the co‐production sessions, provided feedback on the interpretation of the findings, and reviewed and edited the manuscript draft.

## 1 Introduction

There are inequalities in breast cancer outcomes experienced by women of Black African and Black Caribbean heritage in the UK (henceforth, Black women, by which we mean women identifying as Black British, of Black African, Caribbean, or mixed Black heritage). Compared to women of White Caucasian descent, Black women are more likely to present late with symptoms, to be diagnosed with late‐stage and high‐grade breast cancer, and to have lower survival [1, 2, 3, 4, 5, 6]. A range of patient factors are likely to contribute to late symptomatic presentation and hence poorer outcomes: lower breast cancer awareness than White British women [7], lower knowledge of risk factors for breast cancer [8], and lower awareness [9] and lower frequency of practising breast cancer awareness such as breast self‐checks (22% of Black Caribbean and 15% of Black African women vs. 27% of White women) [7]. Emotional and cultural factors, too, can act as barriers to early presentation, for example, fear of a breast cancer diagnosis [10] and cultural stigma [11]. Few breast cancer awareness interventions for Black women have been developed to date, but some have demonstrated increased awareness of breast cancer [12]. The present research aimed to address the gap in behavioural change interventions aimed at improving breast cancer outcomes for Black women in the UK.

### 1.1 Pregnancy as a ‘Teachable Moment’

The ante‐natal period presents an opportunity to increase breast awareness at a younger age before women are eligible for screening (starting at 50 years in the UK) for several reasons. Firstly, most women attend ante‐natal care and are more likely to talk about breast changes than in other contexts. Secondly, pregnancy is a ‘teachable moment’ for healthy lifestyle behaviours [13] when women are likely to engage with health professionals regularly and build up a trustful relationship with them. Third, Black women may be less likely to visit their GP to seek advice about breast changes, especially if the GP is male, but they may be more receptive to breast health information when attending antenatal care, a predominantly female healthcare service. Further, using the appointments with midwives and/or health visitors would be in line with the government policy to ‘*Make Every Contact Count*’ [14]. Therefore, we wanted to explore how we can harness the opportunity of contact with midwives to increase Black women's awareness of breast health and breast cancer.

### 1.2 Co‐Production

Breast cancer interventions are more likely to be accepted if they are developed in collaboration with Black women, including breast cancer survivors [15]. This project adopted a co‐production approach to intervention development, that is, a collaborative way of working where researchers work together with service users and service providers to reach a joint outcome [16, 17]. Our use of co‐production aligned with its definition as a form of equitable and experientially‐informed research [18], whereby the lived experience of service users (Black women with a history of breast cancer, Black women as users of maternity services) was of upmost importance to developing the intervention design brief. Co‐production differs from the method of focus groups as follows: the sharing of power, whereby no perspective is dominant; the development of consensus with regard to the purpose or the end product (e.g., redesign of health services); the continuing dialogue and the joint ownership of key decisions [19]. While the co‐production approach aims to address the power imbalance between service users and healthcare providers, it has been recognised that traditional power hierarchies and socio‐cultural structures can impact the co‐production process [20]. However, it has also been acknowledged that power between co‐production participants should be allowed to fluctuate during the course of a project depending on the what expertise is needed at a particular stage [19].

### 1.3 The Behaviour Change Wheel, the Capability‐Opportunity‐Motivation‐Behaviour (COM‐B) Model, and the Theoretical Domains Framework

In line with previous research emphasising the suitability of the *capability‐opportunity‐motivation‐behaviour* model (COM‐B) [21] for behaviour change during pregnancy [22], we planned the co‐production sessions to address these three dimensions that interact to lead to behaviour change (capability, opportunity, motivation) when considering the key components of a breast cancer awareness intervention and how it should be delivered in ante‐natal settings. We drew on the Behaviour Change Wheel (BCW) and the COM‐B model [21, 23] to focus on modifiable patient factors pertaining to *capability* (e.g., knowledge of breast cancer symptoms) and *motivation* (e.g., beliefs about health consequences of timely help‐seeking). The BCW stages for intervention development [23] (Figure 1) served as a guide for thinking about the target behaviours to address with the intervention, its content, functions, and mode of delivery.

**Figure 1:**
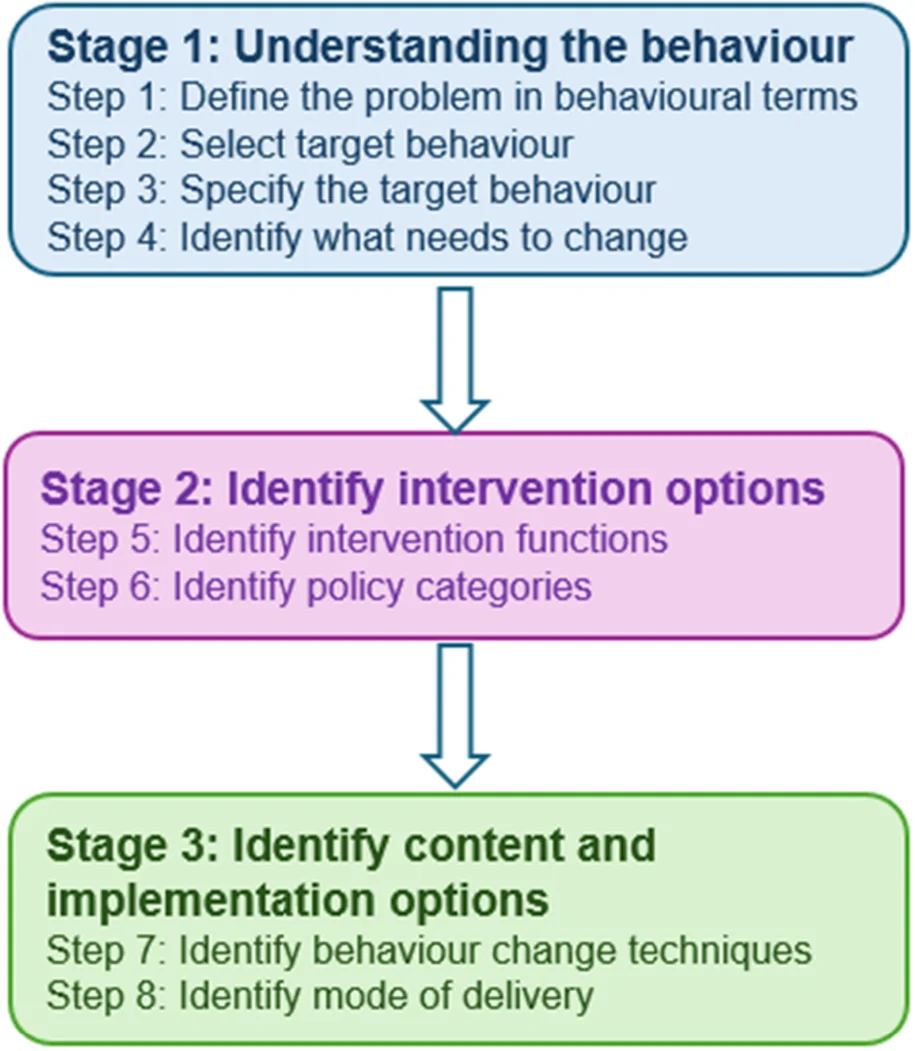
The behaviour change wheel stages for intervention development.

Our approach was further informed by the Theoretical Domains Framework (TDF) [24] which refines the dimensions of *capability*, *opportunity* and *motivation* into 14 domains that relate to key constructs pertaining to health behaviour change (see Table 1). These can be applied in qualitative research to identify barriers and facilitators of health behaviour to help determine how behaviours can be modified [25].

**Table 1:** The theoretical domains framework mapped onto the sub‐constructs of capability, opportunity and motivation from the COM‐B model.

COM‐B	C‐O‐M sub‐constructs	Theoretical domains
Capability	Physical capability	Skills (physical)
	Psychological capability	Knowledge
		Skills (cognitive and interpersonal)
		Memory, attention and decision processes
		Behavioural regulation
Motivation	Reflective motivation	Social/professional role and identity
		Beliefs about capabilities
		Beliefs about consequences
		Optimism
		Intentions
		Goals
	Automatic motivation	Reinforcement
		Emotions
Opportunity	Physical opportunity	Environmental context and resources
	Social opportunity	Social influences

We aimed to co‐produce the design brief for a culturally tailored intervention toolkit to promote awareness of breast cancer among Black women in ante‐natal settings. Our specific objectives were to:

a. Develop the intervention design brief in collaboration with women of Black African and Black Caribbean heritage and with midwives.
b. Determine key behaviours of Black women that could be addressed with the intervention.
c. Assess whether the breast awareness intervention could be acceptable and feasible to Black women and their midwives.
d. Determine the most opportune times for intervention delivery.

## 2 Materials and Methods

### 2.1 Design

The co‐production sessions were informed by the BCW stages of intervention development and by the principles of co‐production, e.g. sharing power, including all perspectives and skills, and valuing the knowledge of all those involved in the research [26, 27]. Four co‐production sessions were conducted with participants from southeast England over the Zoom platform, to facilitate the participation of service users and midwives from various locations. The study received a favourable ethical opinion from the University of Surrey Ethics Committee in June 2022 (reference FHMS 21‐22 166 EGA).

### 2.2 Patient and Public Involvement

A breast cancer survivor of mixed Black African and White heritage acted as PPI (Patient and Public Involvement) representative during the project. She advised on the draft funding application, the ethics documents, and the planning of each co‐production session. She helped recruit participants, facilitated the co‐production sessions, and provided feedback on the interpretation of the findings.

### 2.3 Recruitment Approach

The inclusion criteria for the lay participants were: of Black Caribbean or of Black African heritage, aged 25 or older, with or without a personal diagnosis of breast cancer, resident in southeast England. We recruited women aged 25 or older as these are more likely to experience being diagnosed with breast cancer than women younger than 25 [28]. We purposively recruited women with a history of breast cancer via the patient forum *Breast Cancer Voices* of the charity *Breast Cancer Now*, and via the cancer charity *Black Women Rising*. Four women without a history of breast cancer were recruited from Black African community groups and one, through the PPI representative's social networks. The facilitator was not involved in participant recruitment.

For the midwives, the inclusion criteria were: working in the UK National Health Service (NHS) Trusts in southeast England, with an interest in public health interventions, breast cancer or health inequalities. Personal history of breast cancer was not an inclusion criterion for midwives. They were recruited through snowball sampling, adverts on midwifery groups on Facebook, and professional networks within southeast England.

### 2.4 Procedure

All co‐production sessions were audio‐recorded and transcribed by a professional transcription company. All sessions lasted between 90 and 100 min. The co‐production sessions were jointly moderated by the first author (of Romanian heritage), the PPI representative, and a facilitator (of Indian heritage) experienced in discussing health topics with service users from ethnic minority backgrounds. The PPI representative and the facilitator had rich experience of facilitating groups with people from diverse backgrounds and of participating in projects with qualitative designs. Each session followed a topic guide. In the first co‐production session the participants introduced themselves and described their motivation to take part in the project. The following three co‐production sessions comprised introductions, re‐cap of previous session, brain‐storming exercises, and summary of next steps. Table 2 we provide an overview of the sessions.

**Table 2:** Overview of the co‐production sessions.

Session number and focus	Participants
**Session 1:** The participants introduced themselves and the research team explained the aim of the project and the co‐production approach. Discussion of why breast cancer inequalities exist and why Black women face barriers to breast cancer timely diagnosis. The midwife participants discussed what could be opportune moments to discuss breast health with pregnant women.	1 researcher 1 PPI representative 1 facilitator 3 participants with a diagnosis of breast cancer 5 participants without a diagnosis of breast cancer 3 healthcare professionals (2 midwives and 1 public health apprentice)
**Session 2:** Reminder of the main points of the discussion from session 1. The discussion covered: ‐ what needs to change, on the part of whom (women? healthcare professionals?) to achieve timely diagnosis for breast cancer for Black women ‐ how can ante‐/post‐natal services be harnessed to deliver a breast cancer intervention for Black women? ‐ how should Black women be informed about breast cancer? ‐ what should be the content and format of a resource or intervention toolkit?	1 researcher 1 PPI representative 1 facilitator 4 participants with a diagnosis of breast cancer 4 participants without a diagnosis of breast cancer 3 healthcare professionals (2 midwives and 1 public health apprentice)
**Session 3:** Reminder of the main points of the discussion from session 2. The discussion covered: ‐ the key components of the intervention toolkit ‐ how can ante‐/post‐natal services be harnessed to deliver a breast cancer awareness intervention for Black women?	1 researcher 1 PPI representative 1 facilitator 3 participants with a history of breast cancer 5 participants without a history of breast cancer 3 midwives
**Session 4:** Summary of the main points of the discussion from session 3. The discussion covered: ‐ how the toolkit might look for midwives/health visitors ‐ timepoints when midwives/health visitors might deliver the intervention ‐ support and resources needed by midwives/health visitors to deliver the intervention	1 researcher 1 PPI representative 1 facilitator 3 participants with a history of breast cancer 4 participants without a history of breast cancer 2 midwives

### 2.5 Positionality Statement

The first author is of Romanian heritage, born outside the UK; the second author is a breast cancer survivor of mixed Black African and White heritage; the third and fourth authors are of White British heritage. All four authors have a track record in researching breast cancer inequalities experienced by Black women in the UK, and all authors have experience of conducting qualitative research with Black women on the topic of breast cancer.

### 2.6 Analytic Approach

The first author, an experienced qualitative researcher, led the analysis of the co‐production sessions, with additional input from the PPI representative and the other two co‐authors. We used Template Analysis [29, 30]. Our hierarchical coding template was informed by the BCW three stages and eight steps for intervention development [23] and was applied to all four transcripts as pre‐defined categories to code and organise the data. We excluded Step 6 ‘*Identify policy categories*’ from the coding template because it was not relevant to our research. The analysis was deductive and iterative as each workshop discussion was transcribed, summarised, and shared with co‐production participants after each session.

### 2.7 Data Saturation

As our research consisted of reconvened co‐production sessions with the same, relatively small, group of participants for the purpose of in‐depth deliberation, considerations of data saturation may not be as applicable to our data analysis as they might be to other forms of qualitative research. Instead, the alternative concept of ‘information power’ [31] was applicable to our research. The study had information power because: it had a narrow research question; it had sample specificity, that is, breast cancer survivors and healthcare professionals of Black African/Caribbean heritage; it gathered rich data, and the data analysis was supported by established theory, that is, the BCW. Further, our study achieved ‘meaning saturation’ [32], that is, a richly textured understanding of the issue under research, due to the in‐depth and iterative nature of our co‐production sessions.

## 3 Results

### 3.1 Sample Characteristics

Fourteen women participated in the co‐production sessions: 4 lay women with a personal history of breast cancer, of which one had recent experience of maternity services, 5 lay women without a history of breast cancer, and 5 healthcare professionals (4 midwives and 1 public health apprentice). None of the healthcare professionals had a personal history of breast cancer. Four of the healthcare professionals (HCPs) were of Black African or Black Caribbean heritage, and one was of Asian heritage. All four midwives worked in UK National Health Service (NHS) maternity settings. The age range was 33–60 years, median age = 46 years, see Table 3 with the participants' characteristics. The four participants with a personal history of breast cancer, as well as the PPI representative, had been diagnosed before being eligible for breast cancer screening at ages ranging from 38 to 47. Seven of the 10 participants without a breast cancer diagnosis (five lay women and five HCPs) had either a family member or a close friend diagnosed with breast cancer.

**Table 3:** Characteristics of the participants (*n* = 14).

Participant characteristics	(%)
Background	
Lay participants with a breast cancer diagnosis	4 (28%)
Lay participants without a breast cancer diagnosis	5 (36%)
Healthcare professionals	5 (36%)
Ethnic descent	
Black African	6 (43)
Black Caribbean	5 (36)
Black African & White	1 (7%)
Black Caribbean & White	1 (7%)
Asian	1 (7%)
Born in the UK	
Yes	5 (36%)
No	9 (64%)
Religion	
Christian	10 (72%)
Muslim	1 (7%)
Jewish	1 (7%)
No religion	2 (14%)
Educational attainment	
Degree or higher degree	10 (72%)
Higher education below degree level	1 (7%)
Associate's degree (USA)	1 (7%)
O Level/GSCE	2 (14%)
County of residence or work	
Kent	3 (21%)
Surrey	4 (29%)
Sussex	5 (36%)
London	2 (14%)

We developed three main themes and seven sub‐themes based on the BCW stages and steps of intervention development. Our findings reflected the participants' views on what behaviours should be addressed by the intervention, what the intervention toolkit could consist of, and how it could be delivered in ante‐natal settings. In the quotes below, we indicate participants' age and ethnic heritage, and further add ‘survivor’ if they have a breast cancer diagnosis, or indicate if they are a healthcare professional. Additional quotes from the participants are included in the supporting Information file.

### 3.2 Theme 1: Understanding the Behaviour

#### 3.2.1 Defining the Problem in Behavioural Terms (Step 1)

The first step involves defining the problem in behavioural terms and specifies both the target group and the behaviour itself [23]. The research team explained to co‐production participants that the project aimed to address ethnic inequalities in breast cancer survival and increase early diagnosis among Black women. Participants agreed that these inequalities need to be addressed:

I remember sitting in the chemo chair thinking ‘What can I do?’ because I know I had a very different experience to my [White] mum going through treatment, and I also know that the outcomes for women of colour are harder.

(survivor, 44, Black Caribbean and White)

Participants recognised that Black women's behaviours around breast cancer are problematic because breast cancer (and cancer in general) is a taboo topic in Black African communities. They identified reasons for which Black women tend to present late with symptoms or are reluctant to go for breast cancer screening when invited: lack of trust in the medical profession, cancer stigma, putting faith in God to be protected against ill health, lack of engagement with cancer information that is not culturally appropriate. Participants recognised that these issues need to be addressed through raising awareness and empowering women to navigate the healthcare system:

[I once] joined as an observer a focus group with Black women, and all that you were discussing about inequality and why women don't present, one of the things that came out is a lack of trust of the medical community. People won't present because they don't think they'll be taken seriously, they think they'll have a worse outcome if they go to the GP.

(survivor, 58, Black Caribbean)

#### 3.2.2 Selecting a Target Behaviour (Step 2)

It was agreed that the behaviour needing to be changed was Black women's help‐seeking for breast changes indicative of breast cancer. We did not choose breast cancer screening uptake because Black women are more likely than White women to develop breast cancer prior to being eligible for screening; the median onset age for breast cancer in Black women is 46 years vs. 67 years in White women [33]. Indeed, among our participants, the four breast cancer survivors and PPI representative had been diagnosed at ages ranging from 38 to 47:

I know someone mentioned breast screening, but I think every woman on this call who was diagnosed had their first diagnosis of breast cancer before the screening age. Therefore, we have to really think about how we reach all of those women pre‐screening age, as well as post‐screening age.

(survivor, PPI representative)

#### 3.2.3 Specifying the Target Behaviour (Step 3)

After selecting the target behaviour, the group discussions turned to specifying who needed to perform this behaviour, when, and how often. It was agreed that: pregnant/post‐partum women will need to change their behaviour; they will need to accept information about breast cancer symptoms, breast cancer risks, and how to perform breast self‐checks; they will need to get to know what is normal in relation to their breasts; they will need to engage in body vigilance and check their breasts regularly; and they will need to formulate intentions that they would go promptly to their GP in the event of noticing unusual and persistent breast changes:

Self‐examination. If we can teach ourselves how to examine ourselves even before thinking about approaching any health professionals, it has to come from us.

(Black African, 47)

#### 3.2.4 Identifying What Needs to Change (Step 4)

Participants agreed that change needs to happen among Black women in terms of awareness and behaviour, but also among healthcare professionals who need to be aware that Black women can be at risk of breast cancer before screening age:

In terms of what needs to change, it's twofold. You need to ensure that women at risk are aware of the signs and symptoms and they're signposted where they need to go. […] But maybe it can't just be in isolation of trying to address the awareness amongst women, it's got to be those people that they will go to.[…] Because if women present to their GP and you might have an uneducated GP who doesn't take them seriously, if you happen to be a minority ethnic you might not necessarily get the right support and help.

(survivor, 58, Black Caribbean)

All participants agreed that awareness of breast cancer should be increased, that Black women should be informed that they have a higher risk of developing breast cancer at a younger age, and learn about breast cancer symptoms and risks (COM‐B component *psychological capability*: TDF construct *knowledge*):

It's more about making young Black women know that they have a higher risk factor and that therefore they need to be aware younger.

(survivor, 44, Black Caribbean and White)

Another behaviour seen as facilitating timely help‐seeking was being breast aware and getting to know what constitutes ‘normal’ in relation to one's breasts, for example, shape, size, appearance (COM‐B component *psychological capability*: TDF construct *skills*). By practising breast self‐checks, Black women would be able to notice changes and seek help for these if they do not go away:

We want them to take time to learn their body, take time to get to know what their ‘normal’ body looks like and feels like.

(survivor, 40, Black Caribbean)

Black women should be informed—and persuaded—that detecting breast cancer at an earlier stage leads to better outcomes, and this information should increase their intentions to see their GP promptly (mapped onto the COM‐B component *reflective motivation* and the TDF construct *beliefs about consequences*):

We have to think about how we allow people to know that if you have breast cancer you can survive it, because that's one of the barriers that sometimes stops women from coming forward.

(survivor, PPI representative)

Participants drew attention to the fact that Black women need to be able to relate to and trust the people delivering the breast cancer information (COM‐B component *opportunity*: TDF construct *social influences*) to engage with the advocated preventive behaviours:

It's really important that whoever gives out the information, or if it's an app with someone talking to you, it has to be somebody that looks like us who is in the medical field, so that there is that trust.

(survivor, 44, Black Caribbean and White)

It was recognized that it is not sufficient to know about breast cancer symptoms and perform self‐checks, but to be confident to seek help and to advocate for oneself. Participants argued that Black women should be empowered to seek medical help for breast changes and advocate for themselves (COM‐B component *reflective motivation:* TDF construct *beliefs about capabilities)*:

I don't know whether the two‐week‐wait is really feasible now, but we also spoke about empowering women to know what they're allowed to have. They can have a second opinion, and if they haven't had the referral within two weeks, [shouldn't] feel that they're going to be treated any differently if they ring up or go online with their GP.

(survivor, 58, Black Caribbean)

To increase women's motivation to present promptly to their GP, the intervention should inform them that breast cancer is not a death sentence (COM‐B component *reflective motivation*: TDF construct *beliefs about consequences*):

Many people would need to know the benefits of knowing early. A lot of people think ‘Oh, if I'm diagnosed with cancer, that's it,’ but if we know that if it's caught early then you have better chances, knowing those benefits might help people think a little differently from when they think that once you're diagnosed then that's it.

(Black African, 59)

Overall, the discussions suggested that an intervention delivered to pregnant Black women in ante‐natal settings should increase their intentions (*reflective motivation*) to seek timely help for breast changes in the future. In Table 4 we detail how we mapped the findings against the COM‐B components and the relevant TDF constructs.

**Table 4:** Mapping the behaviour change against the COM‐B components and the TDF constructs.

Target behaviour: Help‐seeking for breast changes indicative of breast cancer			
COM‐B components	TDF constructs	What needs to happen for the behaviour to occur?	Is there a need for change?
*Psychological capability*	*Skills*	Have the physical skills to perform breast self‐checks	Yes, not many Black women may have these skills
*Psychological capability*	*Knowledge*	Know the symptoms and risk for breast cancer	Yes, knowledge of breast cancer is low
	*Knowledge*	Know the correct way to perform breast self‐checks	Yes, not many Black women may have these skills
	*Knowledge*	Know one's family health history in relation to breast cancer	Yes, not many Black women may be aware of the genetic risk of breast cancer
	*Behavioural regulation*	Know how to create ‘if‐then’ rules to seek help promptly if noticing unusual breast changes	Yes, some women may not know the importance of seeking help promptly if noticing breast changes
*Physical opportunity*	*Environmental context and resources*	Be registered with and have access to a GP	Women receiving ante‐natal care are likely to be registered with a GP
	*Environmental context and resources*	Attend ante‐natal appointments	No, it is unusual for pregnant women not to attend their ante‐natal appointments.
*Opportunity*	*Social opportunities and social influences*	Be able to trust the person delivering the breast cancer awareness information	Yes, because women need to relate to the person delivering the information
*Reflective motivation*	*Beliefs about capabilities*	Advocating for oneself	Yes, because not all women may feel confident to present again in primary care if they do not receive a referral to secondary care after first symptomatic presentation
	*Beliefs about consequences*	Hold beliefs that being body vigilant and checking breasts regularly will help notice symptoms of breast cancer on time	Yes, because not all women may be aware of the importance of regular breast self‐checks
	*Beliefs about consequences*	Hold beliefs that seeing GP promptly will help treat breast cancer timely and reduce risk of death	Yes, because not all women may know the importance of early diagnosis
	*Beliefs about consequences*	Hold beliefs that breast cancer is not a death sentence	Yes, because some women may hold fatalistic views about cancer
	*Intentions*	Have established routine of body vigilance and checking breasts for changes	Yes, because not all women practice breast self‐checks

### 3.3 Theme 2: Identifying Intervention Options

#### 3.3.1 Identifying Intervention Functions (Step 5)

The group discussions around the potential content of the intervention, as well as the behaviours it needed to change, revealed three main functions of the intervention: *education* (increasing knowledge of breast cancer and changing women's beliefs about their capabilities), *training* (help Black women develop skills to perform breast self‐checks), and *persuasion* (describe the positive consequences of timely help‐seeking for breast changes in order to increase women's intentions to act upon their symptoms), see Table 5.

**Table 5:** Breast cancer awareness intervention functions.

Target behaviour: Help‐seeking for breast changes indicative of breast cancer		
Intervention function	Definition	Example
*Education*	Increasing knowledge or understanding	Increasing knowledge of breast cancer symptoms Increasing knowledge of breast cancer risks Provide information that it is acceptable to re‐present in primary care or seek second opinion if breast changes persist
*Training*	Imparting skills	Culturally appropriate leaflets on breast cancer providing advice on how to be body vigilant and perform breast self‐checks A film demonstrating breast self‐checks with a Black breast cancer nurse and a Black female actor can help women understand how they should perform breast self‐checks.
*Persuasion*	Using communication to induce positive or negative feelings or stimulate action	Use culturally appropriate leaflets on breast cancer Use a Black female actor to demonstrate breast self‐checks Description of breast cancer symptoms by a Black breast cancer nurse Underline the positive consequences of timely help‐seeking for breast changes

The participants argued that an important function of the intervention should be to empower Black women to navigate the healthcare system and seek second opinions, if necessary, when seeking help for breast changes (COM‐B component *reflective motivation*, TDF domain *beliefs about capabilities*):

The second part about advocacy is knowing what you can do if you get turned down. Because a lot of the issues of late prognosis is because either they've been turned away and said ‘Oh, it's nothing, you're too young,’ but if you're still not happy, knowing that you can advocate for yourself to go and see another GP.

(survivor, 44, Black Caribbean and White)

### 3.4 Theme 3: Design Intervention Content and Implementation Options

#### 3.4.1 Identifying Behaviour Change Techniques (Step 7)

The research team mapped the suggestions from the group discussions onto the taxonomy of behaviour change techniques (BCTTv1) [34]—techniques which specify the active content of behaviour change interventions—and thus identified several techniques that could underpin the breast cancer awareness intervention, see Table 6.

**Table 6:** Suggested behaviour change techniques for the breast cancer awareness intervention.

Target behaviour: Help‐seeking for breast changes indicative of breast cancer		
Behaviour change techniques	Definition	Example
*Credible sources*	Present verbal or visual communication from a credible source in favour of the behaviour	A film featuring a Black breast cancer nurse providing advice and voice‐over, and a Black female actor demonstrating breast self‐checks.
*Credible sources*	Present verbal or visual communication from a credible source in favour of the behaviour	The Black female actor demonstrating breast self‐checks and any other literature on breast cancer should depict Black women with large breasts for greater authenticity.
*Instruction on how to perform a behavior*	Advise or agree on how to perform the behaviour	A film featuring a Black breast cancer nurse providing advice and voice‐over, and a Black female actor demonstrating breast self‐checks.
*Demonstration of the behaviour*	Provide an observable example of the performance of the behaviour, directly in person or indirectly e.g., via film, pictures, for the person to aspire to or imitate	A film with a Black female actor demonstrating how to perform breast self‐checks.
*Information about health consequences*	Provide information (e.g. written, verbal, visual) about health consequences of performing the behaviour	Inform women that seeing a GP about breast changes earlier rather than later can save their life.

Participants suggested that the toolkit should include a video of a Black woman demonstrating how to carry out breast self‐checks, and the information should be delivered by a Black healthcare professional:

If you can do a video of a woman of colour showing how to actually check your breasts, people [will] identify. […] I think they know they should check their breasts but they don't really know how to do it or how frequently, and that would be the first point that I think is really important in the toolkit. […] And I would suggest have a doctor to talk about these things, like a Black oncologist, it's going to be better received if it's somebody of colour that's giving the information.

(survivor, 44, Black Caribbean and White)

Following these suggestions and with further input from the participants, we created a 3‐min film ‘*Love Your Breasts: Touch, Talk, Take Action!*’ with input from a Black female breast cancer clinical nurse specialist (CNS), a professional female Black actor, and an agency specialising in filmed simulations in healthcare settings. The film focuses on being breast aware and practising breast self‐checks, without explicitly mentioning breast cancer. We chose not to use the phrase ‘breast cancer’ to avoid inducing fear and fatalism, as Black women can hold fatalistic beliefs that breast cancer is a death sentence [10]. This pertains to the COM‐B component ‘*reflective motivation*’ and the TDF construct ‘*beliefs about consequences*’. The film emphasises that it is important to get to know what one's breasts normally look like and how they usually feel and to establish a routine of checking one's breasts regularly like once a month, with the model demonstrating self‐checking. The film advises women to make an appointment with their GP if they notice an unusual change that does not go away.

Regarding other potential intervention content, participants suggested that it is important to include information about breast cancer symptoms in a culturally appropriate format, emphasizing that symptoms are not just breast lumps:

Maybe just go for the guidance know your breasts, understanding what's normal for you, check your breasts, look for a lump, anything that's different to you.

(survivor, 58, Black Caribbean)

They also suggested the intervention should contain information specific to Black women about the higher risk of breast cancer before being eligible for screening (from 50 years of age in the UK), statistics on breast cancer incidence in Black women, and description of the importance of timely presentation with symptoms and of early stage diagnosis, as well as advice on how to navigate the healthcare system in the event of experiencing breast changes indicative of breast cancer symptoms, and on how to advocate for oneself if not referred by the GP for a mammography.

#### 3.4.2 Identifying Mode of Delivery (Step 8)

This final sub‐theme reflects discussions around when, how, and who should deliver the breast cancer awareness intervention. There were disagreements as to whether this should be delivered during pregnancy, or only as part of post‐natal care, with some participants expressing concerns that the intervention may not reach Black women who do not have children. An alternative suggestion was for the breast awareness education to be delivered at cervical cancer screening, to include Black women who are not users of maternity services. However, poor engagement with cervical screening services was recognised as a barrier: ‘*It's hard enough for them to want to go for cervical screening*’ (Black African, 59). Various points in the antenatal and postnatal care pathway were considered as opportunities to provide breast health education: booking appointments, antenatal classes, or breastfeeding classes:

If it was something that we could mention in our booking appointment, because the women are more curious about their breasts so they will openly tell you about any changes that they've had, and then we can talk about the breasts and say ‘Have you noticed any changes? If you have, here's a leaflet, or here's a video to watch about the changes in your breasts and what's normal and what's not normal.’

(midwife, 43, Black African)

Some midwives suggested that breast cancer awareness could be integrated into electronic maternity apps like BadgerNet through push notifications:

They have BadgerNotes which is online and the women themselves have their own code to get on. Within BadgerNotes there's the ability to send alerts so that a midwife or healthcare professional [can] have this discussion about education and awareness.

(midwife, 45, Asian)

It was also suggested that midwives could hand out current resources on breast cancer, for example, Breast Cancer Now leaflet ‘*Know your breasts—a guide to breast awareness and screening*’ in a way that minimizes fear and increases receptivity to the information:

Pregnant women could be a bit anxious when they hear that word, but it's important to find a way to say it. It's like being told there is a risk of spina bifida or something like that to a pregnant woman. But it's important to let that woman know there is that risk, and do whatever it takes not to let that happen.

(survivor, 41, Black African)

Participants also identified some potential barriers to intervention delivery, such as Black women's heightened anxiety about pregnancy outcomes given that they are at higher risk of mortality in pregnancy:

If you're a Black African, Black Caribbean woman, you're three and a half times more likely to die in pregnancy, so these groups of women more often than not are coming into a pregnancy having that fear factor already about a pregnancy. Then to try and have a discussion in regards to breast care, what you should look for, I think it's going to be a challenge.

(midwife, 56, Black Caribbean)

It was recognised that midwives will need training not only to deliver the specific breast cancer awareness intervention but to be able to support pregnant/post‐partum women when they experience breast changes:

There needs to be more education for our health professionals. Midwives, we're in the ideal position in those early stages to help support women, give information, but we need to have the correct tools to be able to do that, particularly as a Black midwife, as a Black woman. When I've got another Black woman sitting in front of me and she says to me, ‘I think I've found a lump’ is different to say ‘Is this a lump related to the pregnancy, or is this a lump because of something else?’ and having the confidence to take the conversation forward.

(midwife, 56, Black Caribbean)

## 4 Discussion

This co‐production study aimed to develop the design brief for a behaviour change intervention toolkit to be delivered to Black women in ante‐natal settings. Informed by the Behaviour Change Wheel stages of intervention development [23], and in collaboration with midwives and Black women with and without a diagnosis of breast cancer, we agreed on the key components of a potential breast cancer awareness intervention.

Participants' suggestions for what needs to change on the part of Black women so they can seek help timely for breast changes pertained to the COM‐B domains of *capability* (e.g., skills, knowledge), *opportunity* (e.g., environmental context and resources, social opportunities), and *motivation* (e.g., beliefs about consequences, intentions). The breast cancer survivors in particular advised that Black women should be encouraged to know what is ‘normal’ in relation to their own breasts, be informed that they have a higher risk than White women of developing breast cancer before becoming eligible for screening, and be advised that diagnosing breast cancer at an earlier stage increases chances of survival. These aspects of the breast awareness intervention are in line with other theoretically‐informed UK cancer awareness interventions which have sought to increase symptom awareness, body vigilance, and intentions to seek help in the event of bodily changes which are not normal to self [7, 35, 36, 37]. The current findings are also similar to other cancer awareness interventions co‐produced with ethnic minority groups that have focused on increasing knowledge and intentions, e.g. screening for prostate cancer among Black men in the UK [38].

The findings indicate that the key functions of the breast awareness intervention should be: education, that is, increase Black women's knowledge of breast cancer symptoms (*psychological capability*), training, that is, teach breast self‐checks (*physical capability*), and persuasion, that is, convince that a breast cancer diagnosis is not a ‘death sentence’ and increase intentions to seek timely help (*reflective motivation*). These intervention functions bear resemblance to the functions of other UK breast cancer awareness interventions, e.g. *Promoting Early Presentation (PEP)* [37] which also sought to increase knowledge of breast cancer symptoms and personal risk (through educational messages), ability and confidence to notice changes (through modelling, demonstration, and skills training), and intentions to seek prompt help (through motivational techniques, verbal persuasion, and action planning). The present findings reinforce the evidence base that education, persuasion and training are important functions of breast cancer awareness interventions. However, the present intervention is different from the PEP intervention in that it does not make breast cancer explicit, it is culturally tailored to Black women in the UK, and it is aimed at women not yet eligible for breast screening. Further feasibility testing is needed to determine whether it is acceptable to pregnant Black women and that the functions of education, persuasion and training can be achieved with regard to breast cancer awareness.

As for the key content of the intervention, the participants suggested that a video of a Black woman demonstrating breast self‐checks with advice on how to navigate the healthcare system if noticing unusual breast changes would be particularly educative and impactful. We subsequently produced a culturally appropriate film (‘*Love Your Breasts: Touch, Talk, Take Action*’), but the limited timeframe and financial resources of the project precluded the development of further materials. Instead, the participants' suggested that additional intervention materials could draw on existing resources to accompany the film, for example, the leaflet ‘*Your Breasts During and After Pregnancy*’ produced by the UK breast cancer charity CoppaFeel!. Most importantly, the community members and the midwives recognised the importance of teachable moments and ability to reach a significant number of Black women through antenatal appointments. While some participants recognised that the future intervention may not reach all Black women, for example, those without children, they were nonetheless in favour of the intervention. Discussing the potential drawbacks of the intervention was part of the co‐production brain‐storming activities.

As for the use of the TDF and of the BCW stages for intervention development, this brought structure to the co‐production process and helped identify the key areas for behaviour change. The BCW has been used in conjunction with co‐production approaches in a number of studies [39, 40, 41, 42] and works well with service user‐generated suggestions and preferences for behaviour change interventions. While applying a theoretical lens can make it look as if the findings did not emerge directly from the co‐production discussion, it is worth noting that the analysis and the application of theoretical concepts are research processes that take place *outside* of the co‐production sessions, something that other researchers have noted when applying the Behaviour Change Wheel in co‐production research [40, 42].

### 4.1 Strengths and Limitations

One strength of the present study was the diversity of the Black midwives and Black women included in the co‐production process, in terms of breast cancer diagnosis, ethnic background, religion, and geographical areas within southeast England. The co‐production method and the inclusion of a PPI representative as co‐researcher ensured that the intervention design brief was built with community partnership and developed in line with Black women's lived experiences of breast cancer and of maternity services. The inclusion of NHS midwives enabled us to understand how factors pertaining to pregnant Black women and to midwifery services may pose potential barriers to the delivery of the intervention. By using a co‐production approach we gained insights into how we might assess the acceptability and feasibility of the intervention in the future, similarly to other co‐produced and culturally‐tailored cancer awareness interventions [38]. While assessing the acceptability and feasibility of the intervention was outside the scope of the co‐production project, the feedback from the lay participants highlighted what aspects might make it acceptable to pregnant Black women (e.g., using a Black female actor to demonstrate breast self‐examination).

Another strength is that the healthcare professionals (HCPs), mainly midwives, largely agreed with the lay participants that there are health inequalities affecting Black women and that pregnant Black women would benefit from breast health awareness interventions. As the HCPs were of Black African/Caribbean heritage (except one), the shared cultural heritage may have enabled the participants to build common ground and reach consensus.

As for limitations, holding the co‐production sessions online precluded the use of table‐top activities that might have stimulated more creative thinking about the intervention design. However, holding the meetings online enabled the participants who lived and worked in a very large geographical area to commit to meeting on a regular basis. Another limitation is that while most women in our study had prior experience of using NHS maternity services, we did not include recently pregnant Black women, who might have provided additional insights into the content, acceptability, design, and timing of the intervention. Aware of this, we are planning to refine the present intervention with input from pregnant or recently pregnant Black women and test its acceptability as a part of a follow‐up feasibility study. The use of Template Analysis [29] and of a‐priori, theory‐informed, themes may have constrained the analysis and over‐sensitised the research team to material that could easily fit the stages and steps of intervention development, a potential pitfall of Template Analysis [30]. While not all aspects of the co‐production sessions could be captured by the BCW‐informed template, its use nonetheless ensured a concise summary of the key discussion points relevant to the development of the intervention.

### 4.2 Recommendations for Research and Practice

In terms of the next steps for the intervention toolkit, two steps seem essential: refining the intervention content, and testing it for acceptability and feasibility. Additional content could be created by adapting, or simply using with permission, existing resources tailored for Black women and for pregnant/post‐partum women, respectively. These leaflets could be handed out by midwives (or health visitors) during ante/post‐natal appointments and could accompany relevant conversations about breast changes during and after pregnancy. Furthermore, a script may need to be developed that midwives/health visitors could use to broach the topic of breast health and direct women to the intervention materials.

Regarding the testing for acceptability and feasibility, this is crucial for ensuring that the intervention is acceptable to pregnant Black women as well as to midwives/health visitors, and feasible to deliver in ante‐/post‐natal settings. The feasibility testing will need to take place in ante‐/post‐natal settings and will need to determine that the intervention toolkit does not lead to unintended consequences, for example, increased fear of breast cancer or feeling ‘othered’ in ante/post‐natal healthcare. It would also need to determine the midwives' acceptability of the intervention and the feasibility of incorporating it in usual ante‐/post‐natal care. While delivering breast cancer education in ante‐/post‐natal settings was largely seen as acceptable and feasible by the co‐production participants, some midwife participants cautioned about midwives' potential lack of knowledge of breast cancer and advised that midwives may need adequate training. This echoes past research which showed that sometimes midwives can feel ill‐equipped to deliver health behaviour change interventions on issues other than ante‐/post‐natal care [43]. Further refinement of the intervention is necessary to empower midwives to introduce the topic of breast health at routine midwifery appointments and then direct women to the intervention.

Furthermore, our research suggests that healthcare professionals who have frequent contact with Black women not yet eligible for breast cancer screening, such as midwives and health visitors, should be encouraged to use routine appointments to raise awareness of breast cancer. Similar initiatives have been done in relation to using breast or cervical screening results to include new cancer awareness information [44]. This would be in line with the NHS policy of ‘Making Every Contact Count’ and would capitalise on routine appointments as ‘teachable moments’.

## 5 Conclusion

In the UK there are inequalities in breast cancer outcomes between ethnic groups, with Black women being particularly at risk of late diagnosis and lower survival, compared to White women. Few behaviour change interventions have been designed to date to address these inequalities and to increase Black women's awareness of breast cancer and intentions to seek timely help. This study is the first in the UK to explore how a culturally‐tailored breast cancer awareness intervention could be delivered to Black women in ante‐natal settings to address knowledge gaps and to increase intentions to seek help in the event of breast changes. Our co‐production approach helped determine the design brief of an intervention toolkit to inform Black women about breast health in ante‐natal settings. Further refinement of the intervention and future testing for feasibility and acceptability are needed to ensure the intervention is fit for purpose and acceptable to pregnant Black women.

## Author Contributions

**Afrodita Marcu:** conceptualization, methodology, formal analysis, funding acquisition, project administration, writing – original draft, writing – review and editing. **Lorraine Marke:** conceptualization, methodology, writing – review and editing. **Emma Ream:** conceptualization, methodology, writing – review and editing. **Jo Armes:** conceptualization, methodology, writing – review and editing.

## Ethics Statement

The study received a favourable ethical opinion from the University of Surrey Ethics Committee on 7 June 2022 (Project ID: FHMS 21‐22 166 EGA).

## Conflicts of Interest

The authors declare no conflicts of interest.

## Supporting information


Supporting File

## Data Availability

The authors have nothing to report.
