# Supplementary material for: Co‐Producing the Design Brief for an Intervention to Increase Breast Cancer Awareness Among Black Women in Ante‐Natal Settings: A Qualitative Study With Breast Cancer Survivors, Community Members, and Midwives in the UK
Source: Health Expect. 2026 Oct 16;29(5):e70936. doi: 10.1111/hex.70936 (PMC13674384; doi:10.1111/hex.70936)
Supplement: Supplementary file 1 [file HEX-29-e70936-s001.docx]

**Supplementary table with additional participant quotes**

| **Theme** | **Quotes** |
| --- | --- |
| ***Defining the problem in behavioural terms (Step 1)*** | *At home we don’t speak about screening, breast cancer, cervical cancer. When she received an invite from the NHS to go for the cervical screening, I had to force her to go because everything about her health, she will put it on God; God is with her, it’s a lot about religion and that she’s protected by God, nothing will happen*. (public health apprentice, 33, Black Caribbean) |
|  | *I’ve got two friends who have had breast cancer, and in talking to them and their experiences, there’s something that we realised that we don’t talk about it within our own communities. There is something missing there for Black minority ethnic groups; breast cancer is one of them*. (Black African, 59) |
|  | *Being a Black woman, I would like to know from other Black women why it is that they don’t go for breast screening. My grandmother passed away last year and a couple of years ago she’d made kind of a comment, “Oh, they’ve found a lump, there is a lump on my breast, I need to go for breast screening for breast cancer but I’m not going to go”.* (midwife, 43, Black African) |
|  | *The issue about barriers is so important. So even when we’re talking about breast awareness, it’s not just about being able to find your symptoms, it’s also about feeling confident enough to actually present those symptoms to your doctor or your healthcare professional*. (survivor, PPI representative) |
| ***Identifying what needs to change (Step 4)*** | *How can you infuse information to encourage them to come in earlier?* *So it’s like the leaflet that says* “*If your cancer’s found at Stage 1, your outcomes are this percentage better*”*, and that will maybe encourage them to come earlier. Otherwise, people will think “Yes, I’ve found a lump,” but they still don’t come because they’re scared, so it’s also about educating them to come in earlier and why*. (survivor, 44, Black Caribbean and White) |
|  | *It’s really educating people to empower themselves to get a second opinion, but first of all come in before you need a second opinion*. […] *It’s not just about teaching people how to check yourselves and being aware, but what to do when you come up against problems, because I unfortunately feel that the Black community will come up against problems more often than non-Black people*. (survivor, 44, Black Caribbean and White) |
|  | *I think [community member] has touched on a really important issue, and that’s around the benefits and reducing the fear. I know fear has come up quite a number of times in the conversation. So the toolkit has to somehow reduce the fear of contacting a medical professional, so it’s not just about checking, it’s about that encouragement of that it’s okay to go to a GP or to follow up on what you’ve found.* (survivor, PPI representative) |
| ***Identifying behaviour change techniques (Step 7)*** | *Look at all the resources that are already available – Black Women Rising will have a load of resources, Breast Cancer Care will have resources, the NHS website will have resources although it is a generic site – so whether it’s trying to somehow gather or somehow trying to signpost to those different – obviously getting approvals – but signposting to what’s already available as a start*. (survivor, 58, Black Caribbean) |
|  | *There are apps out there that you can get and download already that will remind you to breast check and they’re all with pink animations representing a White person. So I think maybe the best thing is to reach out to someone that’s already done it and already has an app, and ask them to add on another skin tone or two.* (survivor, 44, Black Caribbean and White) |
| ***Identifying mode of delivery (Step 8)*** | *Maybe when there’s a certain check-up after you’ve got used to being a mum, maybe then, but I wouldn’t want to hear it quite immediately of being… Maybe not even when I’m pregnant because I’d be hormonal and emotional and stuff like that, and then scared and paranoid, so maybe with the healthcare people but maybe at six months or something after*… (survivor, 44, Black Caribbean and White) |
|  | *The other question we ask right from the beginning of pregnancy is about when the last time they had a cervical smear was, so you could do a bit of an integration to say “Look, there’s not just this cancer that we’re interested in, but just be aware,” and then you could then trickle and reinforce as you go through the pregnancy.* (midwife, 48, Black African and White) |
|  | *Depending on the system you use depends on how quickly you can enable changes in it, Clevermed and BadgerNet are very responsive so they all do three-monthly updates. The idea with the push notifications is you have to enable it, so when the women use their app, they have to physically say “Yes, I’m happy to have that done,” otherwise they won’t get any notifications through*. (midwife, 48, Black African and White) |
|  | *It may be a case of reaching out to all the different hospitals etc. and just getting something like that sent to them with a letter that says – or to midwives, I don’t know, whoever you decide to send it to – that if it is midwives, “This should be given out at this time frame blah blah blah,” and if it is female healthcare like when you go for your smears or anything like that, “This should also be given out at that point, specifically for women of colour because they get it at a younger age”*. (survivor, 44, Black Caribbean and White) |
|  | *Once the toolkit’s been designed, you could ask a woman of colour to do the presentation, or even someone who’s a breast cancer survivor because I think it would be more impactful, and I think it would be something that would probably be well-received and I think it’s a really good way of introducing the need of it because a lot of people won’t understand it or believe it*. (survivor, 44, Black Caribbean and White) |
|  | *I think it’s important whatever toolkit that is devised, it’s resourced, but also the midwives feel confident delivering it as well. So definitely trying to get the confidence of the midwives, whether you’re going to pitch it antenatally once or twice and then follow through to the postnatal, or whether you’re going to focus it entirely on postnatal care.* (midwife, 45, Asian) |
|  | *I want to talk about the information about where these guys need to give the information about the breast cancer. I would also agree with them that it’s better to let them be aware during antenatal, because I can remember that most of the local women don’t attend postnatal. So if you have to wait for postnatal, most of them don’t really go back for postnatal and there are still some women that don’t even go for antenatal either*. (Black African, 39) |
